# ForeSight - An AI-driven Smart Living Platform, Approach to Add Access Control to openHAB

**DOI:** 10.1007/978-3-030-51517-1_40

**Published:** 2020-05-31

**Authors:** Jochen Bauer, Michael Hechtel, Christoph Konrad, Martin Holzwarth, Hilko Hoffmann, Thomas Feld, Sven Schneider, Ingo Zinnikus, Andreas Mayr, Jörg Franke

**Affiliations:** 8 Digital Research Centre of Sfax, Sfax, Tunisia; 9 Institut Mines-Télécom, CNRS, Paris, France; 10 Université de Sherbrooke, Sherbrooke, QC Canada; 11 Digital Research Centre of Sfax, Sfax, Tunisia; 12 University of Sfax, Sfax, Tunisia; 13 Institute for Factory Automation and Production Systems, Friedrich-Alexander-University Erlangen-Nürnberg, Egerlandstraße 7-9, 91058 Erlangen, Germany; 14 Deutsches Forschungszentrum für Künstliche Intelligenz GmbH, Stuhlsatzenhausweg 3, 66123 Saarbrücken, Germany; 15 Strategion GmbH, Albert-Einstein-Straße 1, 49076 Osnabrück, Germany

**Keywords:** Architecture, Artificial intelligence, openHAB, Platform, Privacy, Smart home, Smart living

## Abstract

We created an approach for a smart living platform called ForeSight which consists of different modules: a service engineering module, a Web of Things (WoT)-based Internet of Things (IoT) module and an artificial intelligence (AI) component. This paper describes how openHAB, a smart home middleware, is extended to fulfill platform requirements related to a successful interaction with the IoT module of ForeSight, more precisely, to add identity and access management (IAM) to openHAB and comply with European privacy laws.

## Motivation

In recent years, the smart home market has proven its relevance [1, 2]. If all 43 million households in Germany were equipped with smart home technology by 2030 with an average value of 3,000 EUR, this would result in a market potential of 129 billion EUR [3]. For other European countries, the situation seems to be similar [4]. Beyond smart homes, the term “smart living” ranges over various areas that are separated today concerning energy management, health and home automation [5]. The smart home is a core element in a connected world, as well as smart city and smart grid [6]. This will lead to more comfort, better assistance and increased safety and security as well as improved resource efficiency and reduced overall costs. The base for such advanced opportunities is the intense usage of AI.

The ForeSight project follows the approach of an open platform which integrates AI-based solutions, interoperability, context-awareness and established building automation technologies into a flexible multi-domain and multi-component system ranging across different manufacturers and industries [6]. Furthermore, ForeSight will provide the flexibility to add new services and offers corresponding tools for service providers. In Europe, privacy and security issues play an important role [7]. Data needs to be handled carefully. To ensure this, ForeSight will create an adequate IAM mechanism and be as restrictive as possible to ensure privacy concerns, i.e. we will try to keep data stored locally, whenever it is possible.

## State of the Art

**Fig. 1.**
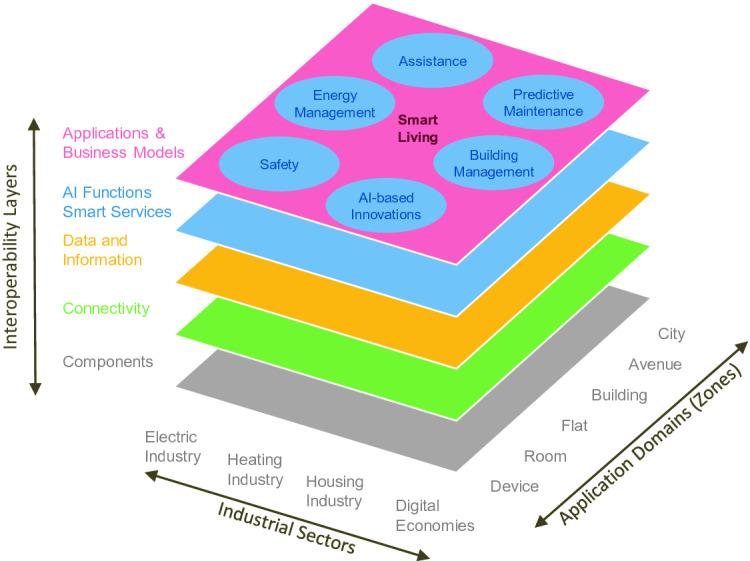
Proposed reference architectural model of smart living.

These days, systems for energy management, classic smart home use cases like lighting as well as health applications exist. To optimize residents’ benefits it is useful to combine these three domains and make them controllable by one single platform, which allows domain and vendor independence. In other areas like manufacturing industry reference architecture models have been established to visualize needs to improve interoperability in general. We adapted these models like RAMI 4.0 [8] to the smart living domain (see Fig. 1). Accordingly, we strive to enable an interoperability level on the business model layer.

After describing necessities of interoperability, privacy and security, we need to introduce the concepts of IAM to ensure a solid base for reaching our goals. There are four topics when it comes to IAM, which need to be considered. In a first step, the user who wants to access the system needs to identify himself (Identification). This claim needs to be verified (Authentication) by the system. Subsequently, it is necessary to grant appropriate rights to the user (Authorisation). For many systems and domains, it is mandatory to log different system events for ensuring auditing, monitoring or tracing (Accountability) [9].

To carry out a target group-oriented authorization, four basic concepts are considered. The first is identity-based access control (IBAC), which provides an access control list for each object, which in turn contains all subjects that are allowed to access the corresponding object [10]. The second concept, role-based access control (RBAC), provides different read and write permissions (generally: transactions) for different user groups [11]. Attribute-based access control (ABAC) defines a similar approach to RBAC, except that users are granted rights based on certain attributes of subjects and objects and environmental conditions [12]. The last type, capability-based access control (CapBAC) turns the rights management the other way around and grants rights based on the token that the user hands over at login [13]. This token then contains an indication of the possibilities a user has on the platform.

openHAB is a smart home middleware, so it is possible to control different systems in one single graphical user interface (GUI) or app. The software uses specific components to offer an abstraction layer for all of its subsystems. To connect to a third-party system like Homematic [14], it is necessary to create a binding. After activating the binding it is possible to search for accessible objects, here for the Homematic bridge and all the Homematic devices, e.g. a switch. The signal of the device and the triggered action from openHAB to the Homematic device is transported through so-called channels. To create a GUI a sitemap is needed. In the sitemap file there is a possibility to name items. An item is a concrete instance of a thing and a channel can be mapped to an item. For automating event-driven tasks there is the concept of rules, a script-like openHAB feature. There are several other smart living middleware systems or promising approaches besides openHAB, for example, universAAL, HomeKit and Connected Home over IP.

## Challenges

To fulfill the idea of our smart living reference architectural model (see Fig. 1) it is necessary to offer a platform architecture which is able to handle upcoming requests as flexible as possible. The corresponding IAM needs to be considered in all systems. This is challenging because existing middleware systems need to be used to connect to different smart home systems to achieve an adequate market penetration. Moreover, as mentioned before, security interferes with comfort, so new concepts need to be evaluated in regard to user acceptance.

openHAB does not yet provide access rights for different user groups and thus does not offer authentication for end-users, besides developer-addressed possibilities. Therefore, openHAB needs to be extended. In addition, there are three variants that allow access from outside on the basis of an encrypted connection. The most secure option is to set up virtual private networks (VPN) to access your system via the router. The second option is to use the specially designed myopenHAB cloud, which can be accessed like various other cloud platforms via a tunnel connection. The third option is to set up a reverse proxy before openHAB, which in turn uses authentication and security certificates to ensure that the smart living system is protected from unauthorized external access. Such remote access strategies are important for several use cases like predictive maintenance scenarios in smart buildings.

## Approach

**Fig. 2.**
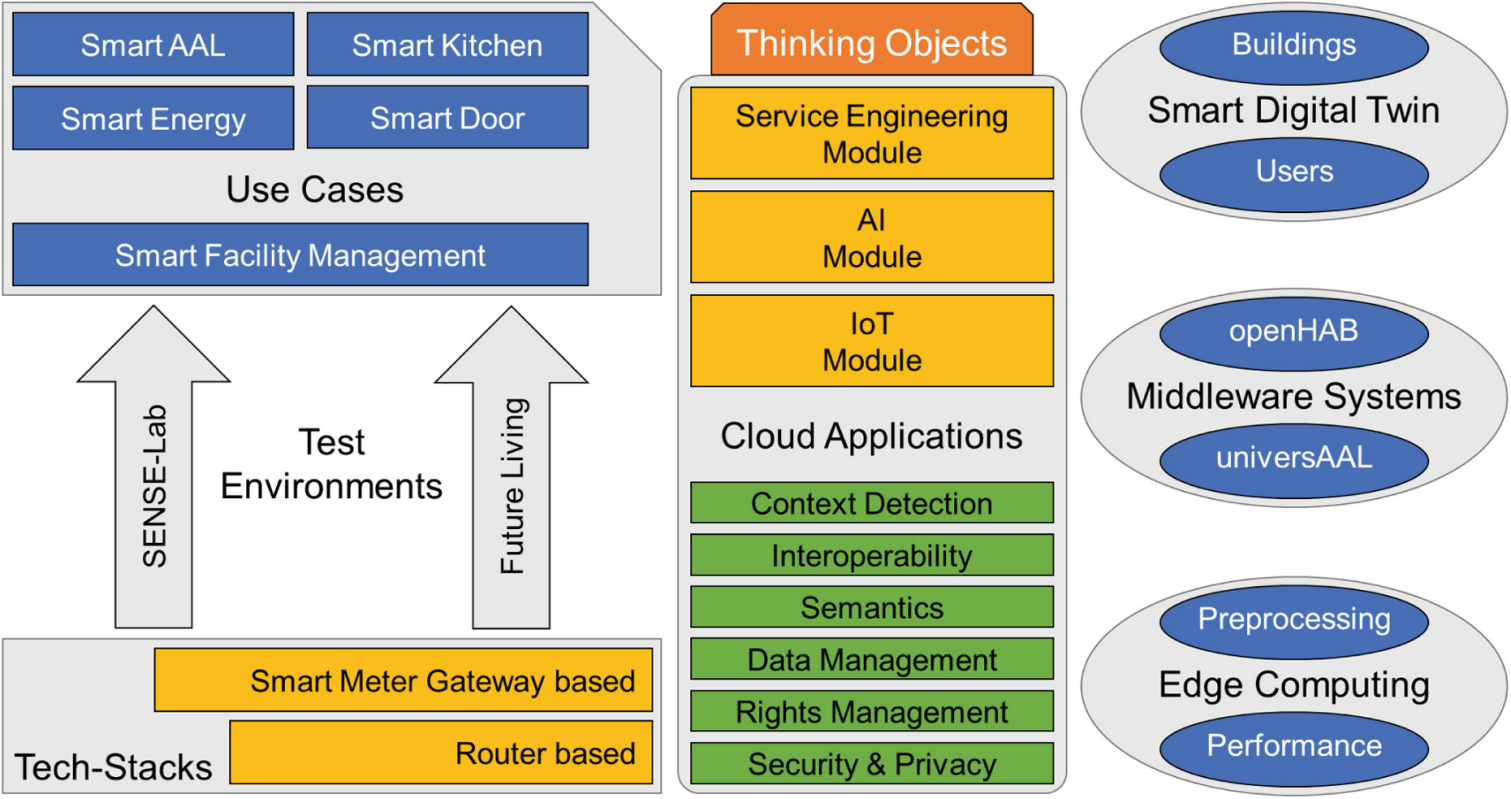
Simplified architecture of the ForeSight Cloud Platform with its three main modules (service engineering, AI module and IoT module).

ForeSight offers a flexible mechanism to handle requests, i.e. it is possible that requests are handled in the local network or, if necessary, will be forwarded to cloud services to increase performance. The core of ForeSight’s architecture approach is the thinking object (TO) - a device or group of devices which offers a specific service to the user or other TOs (see Fig. 2). There are three main modules, which are interacting to fulfill the system needs, here a service engineering module for service providers, e.g. a company of the housing industry, and an AI module to handle requests for computationally intensive operations, e.g. visually based object identification, and an IoT module to connect to different smart home middleware systems, e.g. openHAB, which will be able to connect to many different vendor-specific systems. Summing up, ForeSight is connecting to openHAB to ensure interoperability on a syntactic level. Besides, ForeSight will enable the usage of different smart home middleware systems like universAAL as well.

It will be necessary and helpful for TOs if attributes like context sensitiveness, interoperability, semantic information, data management capabilities, rights management, security and privacy are available. In addition, it is helpful to create a digital twin of the corresponding building and another digital twin of the user to predict their specific behavior. As mentioned before it needs to be considered to handle requests exclusively in the local network. Therefore, we create strategies to adapt cloud-based approaches to edge computing as well, especially methods like preprocessing and device performance enhancements. To verify our concepts different use cases, e.g. smart door and smart kitchen, will be implemented. To prove that our approaches are independent of one technology we are using a minimum of two different technology stacks for each use case, e.g. a common router-based stack (internet router and a smart home middleware), and a smart meter gateway based technology stack (see Fig. 2). First, the use cases will be implemented in laboratories and afterwards in real-world environments like Future Living Berlin [15].

To describe our approach more precisely we want to follow a request through our ForeSight platform. The service provider is a company in the housing industry. When a new tenant rents a flat, a picture and fingerprints of the tenant are captured and this data is stored in a database and transferred to the IoT and AI module, so that the available smart door can be updated with new data. The tenant wants to enter the door and uses his fingerprints and the camera at the door captures a small video sequence. This video is sent to the AI module and the IoT module will receive a reply if the person is verified. To ensure interoperability there is a WoT-based data model available in the IoT module and a corresponding openHAB connection that both systems can communicate with each other to transfer semantic information. Each time there is a data transfer from one module to another module, a privacy and security filter will be applied to ensure that only authorized actions will occur.

This paper focuses on the IoT module, so the AI module and service engineering module will not be described in detail. The AI module of ForeSight offers so-called base services which are important for common AI use cases like object identification. Otherwise, there are complex use cases of the housing industry like a tenant change process. Such use cases benefit enormously by intense AI support. To simplify service engineering for service providers we will offer GUI-driven configuration tools, that companies are able to describe their digital business models easily. The service provider does neither need to consider technical details of the AI module nor the IoT module or the complexity of different smart home middleware systems like openHAB.

**Fig. 3.**
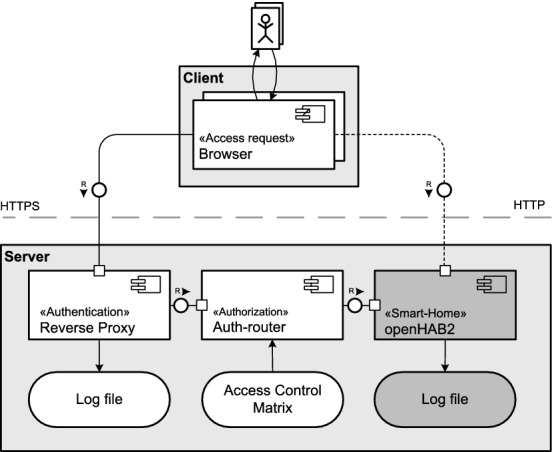
Sidecar approach: Two external tools, here Proxy Server and Auth Router will extend the openHAB environment by enabling relevant services, for example, logging and tracing.

As stated before, openHAB needs to be extended to fulfill an adequate IAM mechanism. In doing so, we decided to use a sidecar approach (see Fig. 3). That means, openHAB is handling its core functions and as a sidecar, we use a proxy server and the tool Auth-router, so common logging functions and configuration options will be available for the system by using these third party tools. The sidecar approach will simplify the consideration of existing systems, for example, logging and tracing.

**Fig. 4.**
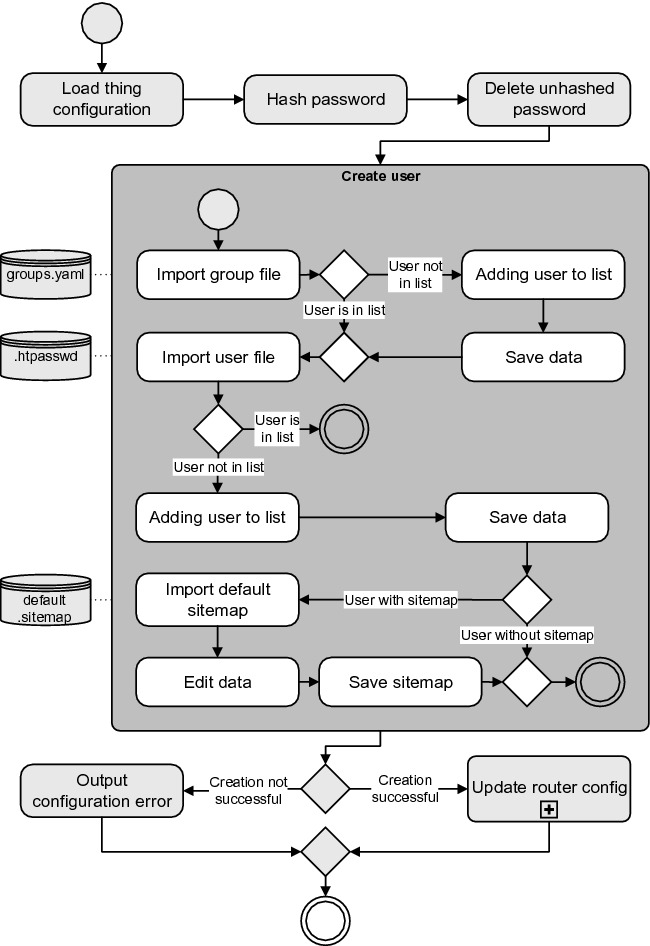
openHAB Auth Binding - create user procedure.

Besides adequate logging functionality, user and group management need to be addressed as well. We decided to use RBAC as a strategy to add IAM to openHAB. RBAC is minimizing configuration effort during the system’s maintenance because necessary changes can be done by changing one specific role or group. This mechanism can be understood by tenants, which is important for accepting such safety-relevant systems in their own home. Furthermore, it is possible to combine this strategy with the sidecar approach. Additionally, RBAC does not need to change openHAB’s core data model, so it is possible to extend openHAB by developing such a binding. We created this binding, which offers Auth-router functionality to the user openHAB’s backend. The procedure for creating a user is shown below (see Fig. 4). For openHAB it is then possible to generate one specific sitemap for each role and so IAM of systems’ resources are ensured. The routing ensures that no user can access sitemaps which are generated for different roles. Our openHAB binding considers user management as well.

ForeSight considers the concept of TOs that combine aspects of three research areas: smart environments (i.e. the physical infrastructure such as sensors, actuators and networks), ambient intelligence (an intelligent network of sensors, radio modules, and computers to proactively but sensibly support people in their lives [16]), AI (agent systems, machine learning techniques). TOs represent physical as well as virtual objects. They aggregate and abstract sensor data of devices to deduce value-added services to users. Several TOs such as sensors, actuators, and lighting in a building can be combined to execute a coordinated activity, e.g. to guide residents through a building.

## Implementation

We developed an openHAB extension to connect to the WoT-based IoT module of ForeSight [17]. This extension will be continuously improved and maintained. Beside the WoT binding we added user management support for openHAB by generating and calling exact one sitemap for each role and restricting the access for non-authorized users. The ForeSight platform with all of its submodules is currently being developed until the end of 2022.

## Discussion

Challenges of semantics and interoperability have long been known in the era of the IoT, especially if different subsystems should work together flawlessly without an overlying de-facto standard. Our approach of trying to offer a WoT-based cloud application, where an AI module and different smart home middleware systems are allowed to connect seems promising for us: We are confident to fulfill all requirements European laws are demanding. Furthermore, we think that it is possible to create a holistic IAM from cloud-driven systems which are often offering CapBAC strategies to smart home middleware systems, that need to be extended in relation to their specific software architectural patterns. Therefore, we need to create strategies to integrate other smart home middleware systems besides openHAB as well, either advanced approaches like universAAL or existing systems like HomeKit or Z-Wave. It is important for ForeSight that lots of existing smart home middleware systems are becoming part of ForeSight, so that ForeSight can achieve its goal to play an important role in the smart living domain.

Extending openHAB by RBAC was helpful to improve access control for tenants in that chosen scenario for a first proof of concept. Hopefully, we will be able to switch from the sidecar approach to a native openHAB solution. Currently, the openHAB developer community seems to work on such strategic ideas for the upcoming major update (version 3.0).
